# Infiltrating immune cells in prostate cancer tissue after androgen deprivation and radiotherapy

**DOI:** 10.1177/03946320231158025

**Published:** 2023-03-06

**Authors:** Ann Erlandsson, Marie Lundholm, Johan Watz, Anders Bergh, Elitsa Petrova, Farhood Alamdari, Thomas Helleday, Sabina Davidsson, Ove Andren, Firas Tarish

**Affiliations:** 1 Department of Urology, Faculty of Medicine and Health, 59566 Örebro University, Örebro, Sweden; 2 Department of Environmental and Life Sciences/Biology, 101086 Karlstad University, Karlstad, Sweden; 3 Department of Medical Biosciences, 377074 Umeå University, Umeå, Sweden; 4 Department of Clinical Pathology and Cytology, 59594 Central Hospital Karlstad, Karlstad, Sweden; 5 Department of Urology, 370894 Västmanlands Hospital, Västerås, Sweden; 6 Department of Oncology-Pathology, Karolinska Institutet, 463758 Science for Life Laboratory, Stockholm, Sweden

**Keywords:** Prostate cancer, androgen deprivation therapy, radiotherapy, immune cells, multiplex IHC

## Abstract

**Objectives:**

Androgen deprivation therapy (ADT) has long been a cornerstone in treatment of advanced prostate cancer (PCa), and is known to improve the results of radiotherapy (RT) for high-risk disease. The purpose of our study was to use a multiplexed immunohistochemical (mIHC) approach to investigate the infiltration of immune cells in PCa tissue after eight weeks of ADT and/or RT with 10 Gy.

**Methods:**

From a cohort of 48 patients divided into two treatment arms, we obtained biopsies before and after treatment and used a mIHC method with multispectral imaging to analyze the infiltration of immune cells in tumor stroma and tumor epithelium, focusing on areas with high infiltration.

**Results:**

Tumor stroma showed a significantly higher infiltration of immune cells compared to tumor epithelium. The most prominent immune cells were CD20^+^ B-lymphocytes, followed by CD68^+^ macrophages, CD8^+^ cytotoxic T-cells, FOXP3^+^ regulatory T-cells (Tregs), and T-bet^+^ Th1-cells. Neoadjuvant ADT followed by RT significantly increased the infiltration of all five immune cells. Numbers of Th1-cells and Tregs significantly increased after single treatment with ADT or RT. In addition, ADT alone increased the number of cytotoxic T-cells and RT increased the number of B-cells.

**Conclusions:**

Neoadjuvant ADT in combination with RT results in a higher inflammatory response compared to RT or ADT alone. The mIHC method may be a useful tool for investigating infiltrating immune cells in PCa biopsies to understand how immunotherapeutic approaches can be combined with current PCa therapies.

## Introduction

Prostate cancer (PCa) is the second most frequently diagnosed cancer in men. Although most PCa patients do not need treatment, it is still the sixth highest cause of cancer-related death in the world.^1^ Androgen deprivation therapy (ADT) has long been an important part of treatment for advanced PCa disease, and in combination with radiation therapy (RT) can improve the treatment of high-risk PCa disease.^2^ The androgen-androgen receptor binding regulates the synthesis of several proteins involved in cell growth, cell proliferation, and repair of DNA double-strand breaks; the last process being particularly important for the improved result when using neoadjuvant ADT combined with RT.^3^ Androgens also have immunosuppressive effects, and so withdrawal of androgen may potentially improve killing of tumor cells by increasing the pro-inflammatory anti-tumor response.^4–6^ A variety of immunotherapeutic approaches have been exploited in PCa treatment but the effects have so far been limited, presumably due to the lack of tumor antigens and the immunosuppressive tumor environment often seen in PCa.^7,8^ The cell damage caused by cancer treatment generally triggers the infiltration of immune cells. Initially, this mainly comprises cells with pro-inflammatory properties; but depending on the actual tumor environment, immunosuppression, repair, and healing processes in which anti-inflammatory cells play an important role can follow. These complex, treatment-triggered inflammatory processes can affect the outcome of the therapy in various ways.^9^

The current overall picture in different cancers, PCa included, suggests that T-bet^+^ Th1-cells, CD68^+^CD163^-^ M1 macrophages, CD8^+^ cytotoxic T-cells, and CD20^+^ B-cells contribute to the anti-tumor immune response, often with improved prognosis as a result.^10–15^ CD68^+^CD163^+^ M2 macrophages and regulatory T-cells (Tregs) expressing transcription factor forkhead box subfamily 3 (FOXP3), on the other hand, are tumor cell supportive due to their ability to suppress the anti-tumor immune response, stimulating cell growth and healing processes as well as impairing treatment effects and prognosis.^16–24^ The immunosuppressive function of FOXP3^+^ Tregs is mediated through their expression of immune-checkpoint inhibitors (ICI). Several ICI blockers have been used to treat different cancers, as have drugs that reduce the number of M2 macrophages, but unfortunately so far with little success in PCa when used as a single treatment.^7,8,23^

Although it is already known that neoadjuvant ADT impairs the ability of tumor cells to repair the DNA damage caused by RT,^3^ a deeper understanding also of the infiltration of immune cells during treatment could contribute knowledge essential for optimizing treatment, perhaps with the inclusion of immunotherapeutic methods.^25^ By using a multiplex immunohistochemical (mIHC) method and digital pathology with multispectral imaging, multiple immune cell markers in the same tissue section can be identified.^26,27^ The mIHC method has therefore been suggested as a tool not only to assess adverse predictors of PCa outcomes, but also to aid in selection of immune therapeutic approaches.^28,29^ A recently published study used mIHC to identify and quantify immune cells after prostatectomy,^15^ but to our knowledge, no study has used mIHC to investigate the infiltration and interrelationship of immune cells in PCa tissue after ADT and/or RT.

The purpose of our study was to optimize an mIHC method for PCa biopsies and use it to study infiltration of T-bet^+^ Th1-cells, CD8^+^ cytotoxic T-cells, CD20^+^ B-cells, macrophages, and FOXP3^+^ Tregs in PCa tissue before and after castration with 8 weeks ADT, RT to a total dose of 10 Gy, or a combination of both treatments.

## Materials and methods

### Tissue sampling and preparation

This study used a previously described cohort of patients^3^ including 48 cases with untreated localized PCa eligible for curative radiotherapy and with the aim to investigate the mechanisms behind the improved treatment effect seen with neoadjuvant ADT. The patients were randomly divided into two trial groups, labeled 1 and 2. Patients in trial group 1 received standard ADT treatment with a GnRH analogue (leuprorelin) followed by RT in daily 2 Gy fractions to a total dose of 87 Gy. Patients in trial group 2 first received RT in 2 Gy daily fractions for five consecutive days followed by GnRH analogue, and then an equivalent higher RT dose to a total of 82 Gy. Biopsies were obtained from all patients before treatment. In trial group 1, a second biopsy was obtained eight weeks after GnRH analogue injection (i.e., when the serum testosterone was reduced to castration level) and a third biopsy setting about three hours after the fifth radiotherapy 2 Gy fraction (total dose 10 Gy). In trial group 2, a second biopsy setting was obtained about three hours after the fifth radiotherapy 2 Gy fraction (total dose 10 Gy); that is, before hormone treatment was initiated. The third biopsy setting from trial group 2 was obtained after hormone treatment and was not analyzed. The study was approved by the Ethical Review Board of Uppsala University (refs: 2011/066, 2011/066/3). We were not able to include clinical data in the analysis because the register for patients included in the cohort ended on 31 December 2020.

### Staining of tissue with hematoxylin-eosin for identification of tumor cell areas

Tissue sections of 4 μm were deparaffinized, stained for 10 min in Mayers HTX (Bio Optica/Dalab, Milano, Italy), rinsed, and stained for 1.5 min in eosin (Histolab AB, Gothenburg, Sweden). The slides were then dehydrated, cleared, and mounted using Tissue-Tek coverslipping film (Sakura Finetek, Torrance, CA). An experienced pathologist blinded to the clinical data marked the tumor area in tissue sections from all biopsies and determined the Gleason score of the untreated tissue. The tissue was graded as follows: 3 + 3 (IUSP grade 1), 3 + 4 (ISUP grade 2), 4 + 3 (ISUP grade 3), 4 + 4 (ISUP grade 4), and 4 + 5 or higher (ISUP grade 5).

### Multiplex immunohistochemical staining

Tissue sections of 4 μm, serially taken after the H&E stained sections, were stained with mIHC using the Opal^TM^ 7 solid Tumor Immunology Kit (PerkinElmer, Waltham MA, USA). In order to optimize the incubation time and concentration of antibodies the staining method was modified from the manufacturer’s instructions, as previously done for colorectal cancer.^26^ For optimization, PCa tissue sections from the actual cohort were used with the aim of allowing exposure times of 30–200 ms and a signal range of 5–30 ms. The sections were dried overnight, heated at 60°C for two hours, deparaffinized, and rehydrated. They were then sequentially stained using specific antibodies directed against T-box expressed T-cells (T-bet) also known as Tbx21 expressed on Th1-cells, CD8, CD20, FOXP3, CD68, and pan-cytokeratin. The nuclear staining was performed with DAPI and visualization of specific antibody binding together with different Opal fluorophores (OF) from the Opal ^TM^ 7 solid Tumor Immunology Kit. The specific antibodies directed against T-bet were used with OF520 (green), those against CD8 with OF570 (red), those against CD20 with OF540 (yellow), those against FOXP3 with OF620 (orange), those against CD68 with OF650 (cyan), and those against cytokeratin with OF690 (magenta). The antibody working concentration and clones were as follows: 4 μg/ml anti-T-bet (clone 4B10: sc-21749, Santa Cruz Biotechnology, Inc, Dallas, Texas, US), 0.12 μg/ml anti-CD8 (clone C8/144B, Dako Agilent, Santa Clara, CA, US), 4 μg/ml anti-CD20 (clone L26 ab9475, Abcam, Cambridge, UK), 0.33 μg/ml anti-FOXP3 (Tregs), 0.24 μg/ml anti-CD68 (clone KP1 M0814, Dako Agilent), and 3.6 μg/ml anti-cytokeratin (pan-CK) for identification of tumor epithelial cells (clone AE1/AE3 M3515, Dako Agilent). Slides were mounted using ProLong Diamond Antifade Mountant (Thermo Fisher, Waltham, MA, USA).

### Multispectral imaging and analysis

The VECTRA 3 Quantitative Pathology Imaging System (PerkinElmer) with standard epifluorescence filters DAPI, FITC, CY3, Texas Red, and CY5 was used for imaging. Initially the whole biopsy was scanned at × 10 magnification, and then the Phenochart software package (PerkinElmer) was used to identify two tumor areas with high infiltration of immune cells. The first tumor area, denoted area 1, was selected to represent the highest infiltration of immune cells in the whole biopsy. The second tumor area, denoted area 2, was selected to represent the area with the highest infiltration of immune cells within 700 μm from area 1. Each area was 669 × 500 μm in size and was scanned at × 20 magnification. A spectral library with imaging collected using the individual dyes and an unstained sample as autofluorescence control was used for spectral unmixing in the inForm software package (PerkinElmer). Before quantitative analysis of each scanned area, 20 representative heterogeneous areas were selected to train machine-learning algorithms for tissue segmentation (differentiation into tumor epithelium and stroma) and cell phenotyping using inForm with the manufacturer’s recommendations. Cell segmentation was based on the nuclear DAPI stain with help from nuclear FOXP3 and T-bet, and membrane CD8, CD20, and CD68 staining.

First, the program was trained to segment the tissue into tumor epithelium area, tumor stroma area, and area without cells. The cells were then classified into the different phenotypes by manually training the program, annotating 50–100 cells identified by each marker. After scanning each image, areas 1 and 2 were manually examined and areas of disinterest such as cellular debris and necrosis were manually drawn and subtracted from the image. Next, inForm was used to calculate the size of the tumor epithelium and tumor stroma tissue area and the number of cell types per mm^2^ tumor stroma and tumor epithelium area of areas 1 and 2. The pathology view tool was used for visual inspection to enable manual calculation of FOXP3 positivity in the tumor cells. An error was made during staining of the marker CD8 in 40 of the untreated tissue biopsies, and so the result for CD8 in the untreated tissue is based on only eight samples.

### Staining of tissue with anti-CD163

Sections of 4 μm tissue taken serially after the mIHC stained section were deparaffinized followed by antigen retrieval in EnVision FLEX Target Retrieval Solution, high pH (Dako, Glostrup, Denmark) using PT-link at 97°C for 20 min. Slides were then incubated for 30 min at room temperature with the monoclonal mouse anti-human-CD163 antibody (clone 10D6, 1:200, Novocastra, Leica Microsystems, Newcastle, UK). The immunohistochemical EnVision visualization system was used with the standard method of horseradish peroxidase and 3,3’-diaminobenzidine, incubating the sections with a dextran polymer conjugated with secondary antibodies for 20 min and substrate working solution FLEX DAB sub-chromophore for 5 min in Autostainer Link 48 according to the manufacturer’s instructions (Dako). Counterstaining was performed using Mayer’s hematoxylin, and slides were dehydrated, cleared, and mounted using Tissue-Tek coverslipping film (Sakura Finetek). Tonsil tissue was used as a positive control for the CD163 antibodies. A person with experience in assessing CD163 stained cells inspected the whole biopsy and graded the tissue as (a) 20% or less of the stroma cells expressing CD163 and (b) more than 20% of the stroma cells expressing CD163. In cases where there were grading difficulties, an experienced pathologist was consulted.

### Statistical methods

A linear mixed model on arcsine square root-transformed proportions (tumor epithelium area/[tumor epithelium area + tumor stroma area]) was used to confirm that areas 1 and 2 were selected similarly in the biopsies from the different treatment groups. We then analyzed areas 1 and 2 separately. Differences between treatments (before, ADT, RT, and ADT + RT), tissues (tumor stroma and tumor epithelium), and their interaction effect on cell density were tested using generalized mixed linear models. The data were based on cell counts, and so we used a negative binomial distribution for the log link function. For the parameters with repeated measures (tissue: tumor stroma and tumor epithelium from the same sample; treatment: before – ADT – ADT + RT and before – RT, respectively, from the same patient), we specified the structure of the covariance matrix as compound symmetry. To test whether FOXP3 expression in tumor cells (a 0/1 variable) was different between treatments and tissue, we used a binomial log link function along with the covariance matrix structure specified as compound symmetry for the repeated measures variables. When there was a statistically significant effect (α = 0.05), we used post-hoc pairwise comparisons to test which treatments differed.

We used Spearman rank correlations to test if there was a relationship between the Gleason score and the cell density before treatment for each cell type (α = 0.05). In addition, we explored the cell data to investigate potential relationships among the densities of different cell types using Spearman rank correlations stratified by treatment, tissue and area. To account for multiple comparisons in these additional analyses (240 correlations in total), we used α = 0.001. Data were analyzed using SPSS v.22 (IBM, Armonk, NY, USA) and STATA release 14 (Stata Corp., College Station, TX, USA).

## Results

### Selection of areas analyzed

A previous study on the same cohort demonstrated that 7% of cells were Ki67 positive before treatment, that castration reduced this proportion to 1.5%, and that five days of radiotherapy in both arms further reduced it to 0.5%.^3^ Our study focus was to select biopsy areas with tumor cells and high infiltration of immune cells. The analysis using a linear mixed model on arcsine square root-transformed proportion (tumor epithelium area/[tumor epithelium area + tumor stroma area]) verified that there was no significant treatment effect on the proportion in the actual areas selected for analysis in our study (the structure of the covariance matrix specified as compound symmetry for the repeated measures variable was F = 2.546, df = 3, 91.85, *p* = 0.061 for area 1; and F = 2.106, df = 3, 88.16, *p* = 0.105 for area 2).

### Analyses of immune cell subsets in PCa tissue before and after treatment

**Figure 1.**
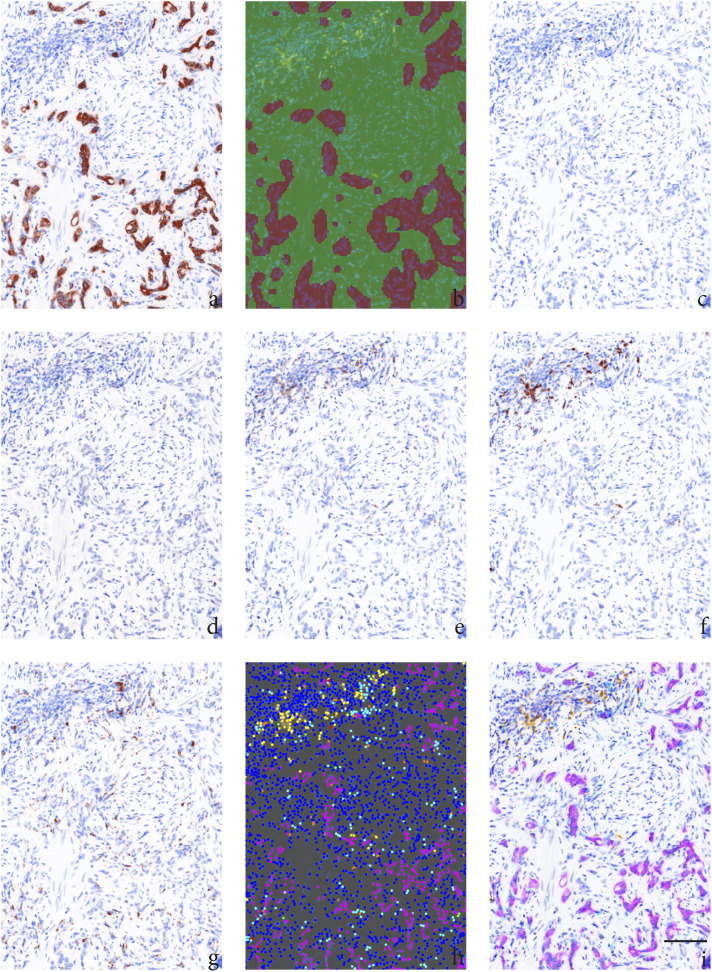
Example of an area 1 with a high number of infiltrated immune cells from a PCa patient (case A) treated with neoadjuvant ADT followed by RT. The tumor epithelium area, tumor stroma area, and different cell types were visualized using inForm software and the pathology view tool. (a) Cytokeratin staining of cytoplasm in tumor cells, (b) areas calculated as tumor epithelium area (brown) and tumor stromal area (green), (c) FOXP3 nuclear staining of Tregs, (d) T-bet nuclear staining of Th1-helper cells, (e) CD8 staining membranes of cytotoxic T-cells, (f) CD20 staining membranes of B-cells, (g) CD68 staining membranes of macrophages, (h) composite image demonstrating each machine-calculated cell as a colored dot: T-bet expressing cells (green), CD8 expressing cells (red), CD20 expressing cells (yellow), FOXP3 expressing cells (orange), CD68 expressing cells (cyan), tumor epithelial cells expressing cytokeratin (magenta), and other stroma cells (only DAPI stained blue), (i) composite image demonstrating all six antigens: T-bet (green), CD8 (red), CD20 (yellow), FOXP3 (orange), CD68 (cyan), cytokeratin (magenta), and blue DAPI staining of nucleus. Magnification × 20, scale bar 100μm.

**Figure 2.**
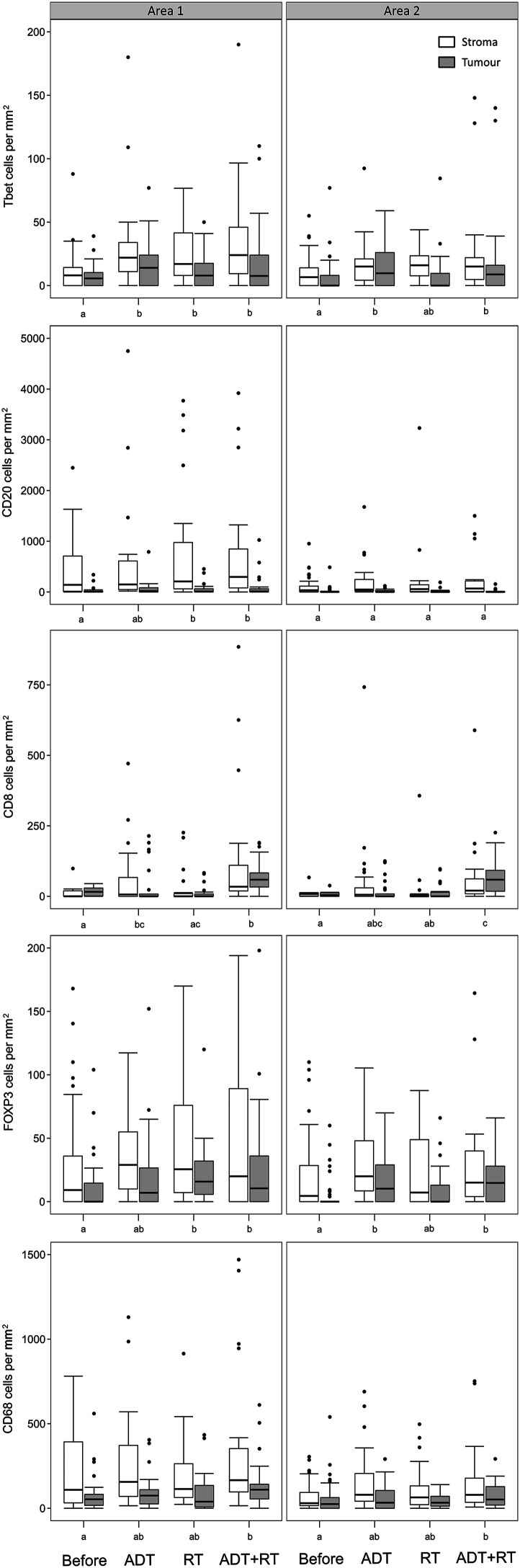
Boxplots showing the density of different immune cells in PCa tissue before (*n* = 48) and after treatment with androgen deprivation (ADT; *n* = 25), radiotherapy (RT; *n* = 25), and ADT followed by RT (*n* = 23). White boxes represent densities in the tumor stroma area and gray boxes the tumor epithelium area. Left panels show the densities in the areas with the highest infiltration of immune cells (Area 1) and right panels an adjacent area (Area 2). Within panels, treatments that do not share the same letter (a – c) have statistically different cell densities.

We investigated the infiltration of immune cells in PCa tissue before and after castration with 8 weeks ADT, RT to a total dose of 10 Gy, or a combination of both treatments. Clinical data for the 48 patients included in our study (Table 1) were extracted from a previously published study.^3^ We used mIHC staining, spectral imaging, and a pathology view tool to visualize and quantify the number of T-bet^+^ Th1-cells, CD20^+^ B-lymphocytes, CD8^+^ cytotoxic T-cells, FOXP3^+^ Tregs, and CD68^+^ macrophages (Figure 1). As expected, immune cell infiltration was more prominent in tumor stroma than in tumor epithelium, and more prominent in area 1, which was selected to represent the highest immune infiltration, than in the adjacent area 2 (Figure 2, Table 2). There is a significant overall effect of treatment and tissue areas (tumor or stroma) on the cell densities, with the exception for treatment and CD20 positive cells in Area 2 and the effect of tissue type on CD8 positive cells (Table 2). No effect of treatment or tissue type on the expression of FOXP3 in tumor cells could be seen (Table 2). The statistical analysis of the effect of the different treatments on the density of the different cell types was carried out using pairwise post-hoc comparisons (Figure 2). In comparison to the untreated tissue, all three treatments increased the infiltration of three or five of the analyzed immune cells, with a significantly increased infiltration of T-bet^+^ Th1-cells, and FOXP3^+^ Tregs in area 1 and/or 2 (Figure 2).

**Table 1.** Age, Gleason score at diagnosis, serum PSA, and testosterone in the two treatment arms at diagnosis and after treatment.

	ADT, ADT + RT *N* = 25	RT *N* = 23
Age in years, median (range)	70 (56–78)	69 (55–78)
Gleason score (%) at diagnosis based on all 6–12 biopsies	6 (4%) 7 (60%) 8 (12%) 9 (24%)	6 (8%) 7 (65%) 8 (8%) 9 (17%)
Serum PSA in ng/ml, median (quartiles)		
At diagnosis	11 (7.6–19.5)	8.3 (3.9–15)
After ADT	0.8 (0.36–1.6)	—
After RT	—	9.5 (4.7–19.25)
After ADT + RT	1.2 (0.34–2.2)	—
Serum testosterone in nmol/L, median (quartiles)		
At diagnosis	Not determined	Not determined
After ADT	0.7 (0–0.95)	—
After RT	—	9.4 (7.8–12.5)
After ADT + RT	0.5 (0–0.93)	—

**Table 2.** Overall main effect of treatment and tissue on the different cell types or FOXP3 expression in tumor cells. The result is generated from generalized linear models testing the effects of treatment (before, ADT, RT, and ATD + RT) and tissue (stroma and tumor). The upper panel shows the densities of different cell types in areas 1 and 2 using a negative binomial link function. The lower panel shows the FOXP3 expression in tumor cells (yes/no) using a binomial link function.

Source of variation		Cell marker in area 1			Cell marker in area 2		
		T-bet			T-bet		
Upper panel		*F*	*d*	*p*	*F*	*df*	*p*
	Treatment	12.2	3, 34	**< 0.001**	5.8	3, 234	**< 0.001**
	Tissue	13.7	1, 234	**< 0.001**	2.7	1, 234	0.1
	Treatment × tissue	0.3	3, 234	0.83	0.3	3, 234	0.84
		CD20			CD20		
		*F*	*d*	*p*	*F*	*df*	*p*
	Treatment	5.3	3, 234	**< 0.001**	12.2	3, 234	0.75
	Tissue	107.1	1, 234	**< 0.001**	13.7	1, 234	**< 0.001**
	Treatment × tissue	1.2	3, 234	0.32	0.3	3, 234	0.54
		FOXP3			FOXP3		
		*F*	*d*	*p*	*F*	*df*	*p*
	Treatment	5.3	3, 234	**< 0.01**	5.3	3, 234	**< 0.01**
	Tissue	16.6	1, 234	**< 0.001**	14.0	1, 234	**< 0.001**
	Treatment × tissue	0.3	3, 234	0.81	1.3	3, 234	0.29
			CD8			CD8		
*F*			*d*	*p*	*F*	*df*	*p*
	Treatment	6.2	3, 150	**< 0.001**	12.2	3, 150	**0.02**
	Tissue	2.6	3, 150	0.11	13.7	3, 150	0.28
	Treatment × tissue	0.1	3, 150	0.94	0.3	3, 150	0.63
		CD68			CD68		
		*F*	*d*	*p*	*F*	*df*	*p*
	Treatment	3.7	3, 234	**0.01**	2.8	3, 234	**0.04**
	Tissue	43.1	1, 234	**< 0.001**	18.4	1, 234	**< 0.001**
	Treatment × tissue	0.1	3, 234	0.97	1.4	3,234	0.26
Lower panel		FOXP3 tumor cells, areas 1+2		
		*F*	*d*	p
	Treatment	1.5	3, 117	0.22

*F*: F-value, *df*: degree of freedom, *p*: significance.

After neoadjuvant ADT followed by RT, all five cell types increased significantly in area 1, while in area 2 a significant increase of cells expressing T-bet, FOXP3, CD8, or CD68 was observed (Figure 2). After ADT, there was a significant increase of cells expressing T-bet in both areas, a significant increase of cells expressing CD8 in area 1, and a significant increase of cells expressing FOXP3 in area 2 (Figure 2). After RT, there was a significant increase of cells expressing T-bet, CD20, or FOXP3 in area 1 (Figure 2). Collectively, these results demonstrate that neoadjuvant ADT treatment in combination with RT treatment contributes to higher immune infiltration in the prostate compared to single ADT or RT treatment.

**Figure 3.**
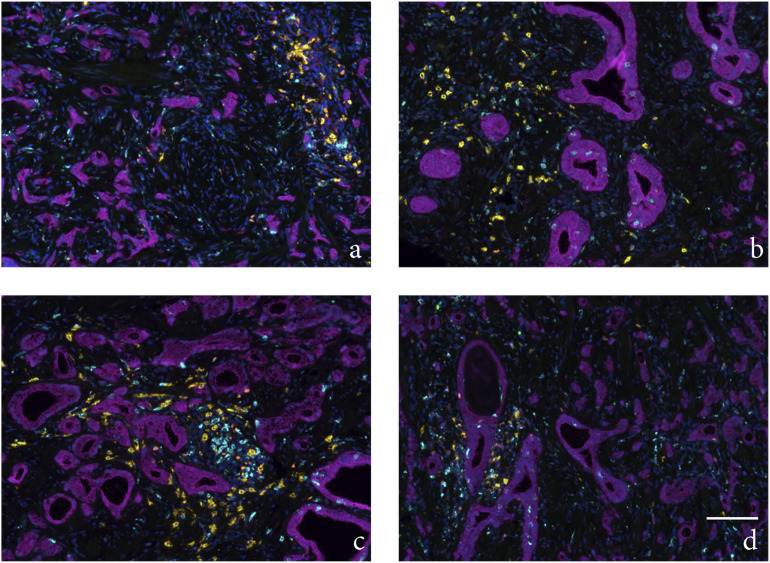
Representative multispectral imaging of immune cell infiltrate in areas with high inflammation (area 1) after treatment, all demonstrating high numbers of CD20 (yellow) and CD68 (cyan) stained inflammatory cells. (a) Case A tissue after ADT followed by RT, (b) case B tissue after ADT, (c) case C tissue after RT, (d) case D tissue after ADT. Magnification × 20, scale bar 100 μM.

The microscopic appearance of the tumor cells and immune infiltrate was, as expected, very heterogeneous in the PCa tissue. However, the tumor stromal tissue of area 1 contained particularly high concentrations of CD20^+^ B-lymphocytes (500–1000 cells/mm^2^) and CD68^+^ macrophages (250–500 cells/mm^2^), followed by lower concentrations of CD8^+^ cytotoxic T-cells (50–100 cells/mm^2^), FOXP3^+^ Tregs (30–80 cells/mm^2^), and T-bet^+^ Th1-cells (10–50 cells/mm^2^) (Figures 2 and 3).

### Expression of FOXP3 in PCa tumor cells

**Figure 4.**
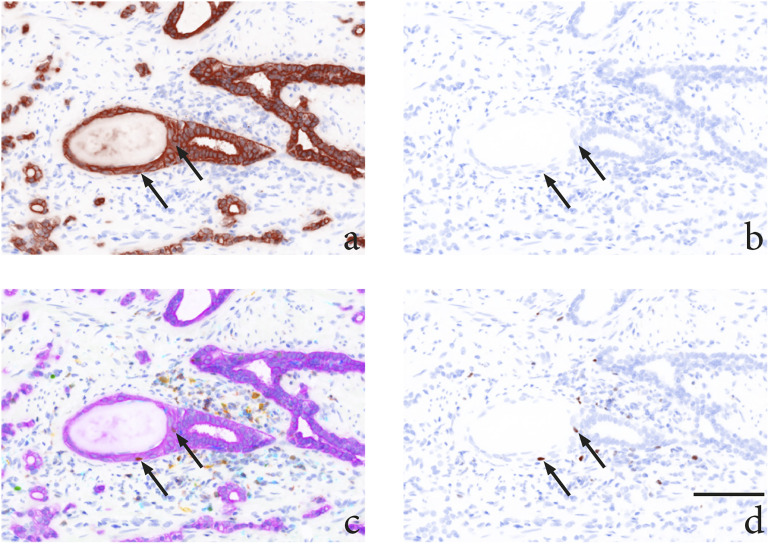
Tissue from a patient (case D) treated with ADT with black arrows pointing at tumor epithelial cells expressing FOXP3. (a) pan-CK staining visualizing tumor cell cytoplasm, (b) DAPI staining visualizing nucleated cells, (c) composite image, (d) FOXP3 staining. Magnification × 20, scale bar 100 μM.

By visual inspection, we could also identify tumor cells that were double stained for cytoplasmic cytokeratin and nuclear FOXP3 (Figure 4). There was a slight increase in the proportion of cases with FOXP3 expression in the tumor cells after treatment; 32% of cases treated with neoadjuvant ADT and RT showed FOXP3 tumor cell positivity, compared to 17% of untreated cases (Table 3). However, differences between treatments and tissue were not statistically verified.

**Table 3.** Gleason score and CD163 and tumor cell FOXP3 expression analysis in areas 1 and 2, analyzed in the same biopsy used for mIHC.

Analysis	Before treatment N = 48	Arm 1, ADT N = 25	Arm 1, ADT + RT N = 25	Arm 2, RT N = 23
Gleason score, ISUP grade 1–5 (number of cases, %)	1 (23, 48%) 2 (8, 17%) 3 (8, 17%) 4 (4, 8%) 5 (5, 10%)			
≤ 20% CD163^+^ cells (a), > 20% CD163^+^ cells (b), (number of cases, %)	a (12, 25%) b (36, 75%)	a (3, 12%) b (22, 88%)	a (2, 8%) b (23, 92%)	a (1, 4%) b (22, 96%)
FOXP3 expression in tumor cells, yes or no in area 1 (number of cases, %)	Yes (8, 17%) No (40, 83%)	Yes (3, 12%) No (22, 88%)	Yes (8, 32%) No (17, 68%)	Yes (6, 26%) No (17, 74%)
FOXP3 expression in tumor cells, yes or no in area 2 (number of cases, %)	Yes (4, 8%) No (44, 92%)	Yes (7, 28%) No (18, 72%)	Yes (3, 12%) No (22, 88%)	Yes (3, 13%) No (20, 87%)

### Relationships between infiltrated immune cell subsets and associations with Gleason score

We found a positive significant correlation between immune cells, particularly in the tumor stroma, from both treated and untreated tissue (Table 4). Moreover, as expected, there was a positive correlation between Gleason score and the number of CD68^+^ cells/mm^2^ tumor stroma in both area 1 (*p* = 0.023) and area 2 (*p* = 0.002) of the untreated tissue. None of the other immune cell subsets analyzed were correlated with Gleason score in the untreated tissue.

**Table 4.** Significant (*p* ≤ 0.001) correlations between cell types in tumor and stroma tissue of areas 1 and 2.

Area and tissue analyzed	Before treatment *N* = 48	Arm 1, ADT *N* = 25	Arm 1, ADT + RT *N* = 25	Arm 2, RT *N* = 23
Area 1, stroma	FOXP3 and CD68; r = 0.50 FOXP3 and CD20; r = 0.61 CD68 and T-bet; r = 0.46 CD68 and CD20; r = 0.45	CD68 and T-bet; r = 0.73	FOXP3 and T-bet; r = 0.60 FOXP3 and CD20; r = 0.66 CD68 and T-bet; r = 0.60 CD68 and CD8; r = 0.67	
Area 1, tumor		FOXP3 and Tbet; r = 0.64 CD8 and Tbet; r = 0.64		CD68 and CD8; r = 0.70
Area 2, stroma	FOXP3 and CD68; r = 0.48	CD68 and T-bet; r = 0.62 CD68 and CD8; r = 0.62 CD68 and CD20; r = 0.62		
Area 2, tumor				FOXP3 and CD68; r = 0.63 FOXP3 and CD8; r = 0.67 CD68 and CD8; r = 0.70

r: correlation coefficient.

### Expression of CD68 and CD163

To investigate whether the tumor-associated macrophages which were positively stained for CD68 in the mIHC method were likely to be of an M2 phenotype (i.e., stained for both CD68 and CD163), we used a standard IHC method to semi-quantify the amount of CD163^+^ cells and visually inspected the serially CD68 and CD163 stained sections. In the untreated cases, 75% had a high proportion (> 20%) of stroma cells expressing CD163, while after treatment this rose to 88–96% of cases (Table 3). Although not stained in the same tissue section, it was apparent that the number of cells stained with CD163 was higher than the number of cells stained with CD68 in a majority of the prostate biopsies (Supplemental Figure 1). This supports the assumption that the majority of tumor-associated macrophages with CD68 positivity are of an M2 phenotype also expressing CD163.

## Discussion

We have previously demonstrated that neoadjuvant ADT impairs the cell’s ability to repair DNA double-strand breaks caused by ionizing radiation during RT.^3^ In this study, we demonstrated that neoadjuvant ADT in combination with RT also results in significantly increased infiltration of Th1-cells, cytotoxic T-cells, B-cells, Tregs, and macrophages. ADT alone significantly increased the numbers of Th1-cells, cytotoxic T-cells, and Tregs, whereas RT alone increased the numbers of Th1-cells, B-cells, and Tregs.

The increased numbers of pro-inflammatory T-bet^+^ Th1-cells observed for all three treatments in this study is likely an effect of the tissue damage and elevated levels of antigens from damaged cells. Few studies have investigated the impact of these cells in PCa, but one study indicates that T-bet^+^ Th1-cells could reduce the metastasis rate and another suggests that T-bet^+^ Th1-cells can enhance anti-tumor responses in hepatocellular carcinoma.^10,14^ Increased infiltration of FOXP3^+^ Tregs was also seen after all three treatments; this might reflect efforts by the tissue to reduce the pro-inflammatory reactions and mitigate the damage, which if occurring during therapy could contribute to a reduced effect of the treatments.^20,23^

Androgens have several immune suppressive effects, and withdrawal of androgen therefore favors the infiltration of pro-inflammatory cells. Our results demonstrated a higher infiltration of not only T-bet^+^ Th1-cells, but also CD8^+^cytotoxic T-cells after ADT alone as well as after ADT combined with RT. Moreover, RT or neoadjuvant ADT followed by RT increased the number of CD20^+^ B-cells. In line with this result, it has previously been shown that a dose of 10 Gy can induce the expression of CD20 surface receptor on B-cells.^30^ High numbers of cytotoxic T-cells (CD8^+^ T-cells) have been linked to improved survival in PCa^13,15^ and enhancing the function of the CD8^+^ T-cell has been suggested as a therapeutic application.^31^ A recent study using neoadjuvant treatment with the anti-CD20 antibody rituximab indicated some advantage in treatment of high-risk PCa.^20^ However, the role of infiltrating CD20^+^ B-cells in PCa during RT treatment has not been explored, and further investigations are needed before we can draw any conclusions about the actual effect of an increase in CD20^+^ B-cells during RT.

There are two functionally different main subtypes of macrophages: the pro-inflammatory M1 macrophage and the anti-inflammatory M2 macrophage. CD68 is expressed on the membrane of both M1 and M2 macrophages, and the marker CD163 has been used to identify tumor-associated macrophages (TAM). It has previously been shown that the majority of the TAM present in PCa tissue is of an M2 phenotype.^18,19,32^ After neoadjuvant ADT combined with RT, a significant increased infiltration of CD68^+^ TAM was demonstrated using the mIHC method. Standard IHC staining showed that 75% of untreated biopsies and 92% of cases treated with neoadjuvant ADT combined with RT had a high infiltration of CD163^+^ cells. The staining of CD68 and CD163 was not done in the same tissue section or with the same staining method, but the visual inspection indicated that the number of CD163^+^ cells was higher than the number of CD68^+^ cells in all of the biopsies. It is therefore likely that the majority of the infiltrating CD68^+^ macrophages in our study are of an M2 phenotype. Monocytes mainly differentiate into macrophages, but can also differentiate into fibroblast-like cells that are able to express CD163.^33^ Thus, we cannot exclude infiltration of CD68^-^ and CD163^+^ cancer associated fibroblasts. In comparison to M1 macrophages, M2 macrophages are anti-inflammatory, repairing, and tumor-promoting, properties that are advantageous to a damaged and stressed tissue. The actual increase of macrophages seen after neoadjuvant ADT combined with RT could be related to the higher damage caused by this treatment and the greater need to repair the damage. It has recently been demonstrated, by another study, that the intraprostatic immune environment two weeks after RT is dominated by myeloid cells, that is, macrophages.^34^ In this context, we have to point out that the biopsies taken after RT and ADT + RT is capturing the acute inflammatory effects after five days of RT. The biopsy taken after RT followed by ADT is not analyzed in our study, but could have given us more information of the long term effect of RT. Further investigation is needed to determine how macrophages influence the effect of the neoadjuvant ADT and RT therapy, and whether interventions that reduce the number of macrophages of the M2 phenotype can be added.^34–36^

The most important strength of our study is that we used mIHC and studied the infiltration of immune cells before and after treatment in PCa tissue from the same patient. This design is not widely used due to the ethical dilemma of justifying obtaining a biopsy from a patient during ongoing treatment. The study cohort included 48 cases divided into two treatment arms, and we analyzed two selected areas in one biopsy from each case and treatment. In total, 121 samples were collected from these 48 participants, and hence, we used a repeated measures design in which multiple sampling was carried out before and after treatment. Despite a strict selection of patients with locally advanced PCa and biopsy sampling from palpable tumor areas it is always a question as to whether or not the persons participating in the study could represent a larger population. The statistical model used incorporates the sampling error in the error term, together with the “natural” variation among the statistical units, that is, persons participating in the study and regardless of low sample size and the use of only two Areas in one biopsy, we found many statistical significant effects in our analyses. We focused on tumor areas with high immune cell infiltration, in order to make the area selection as simple and equal as possible between the different biopsies. However, it is important to point out that it can be more difficult to select a tumor area correctly in tissue affected by cancer treatment, because the treatment can lead to collapsed and irregular glands even in benign areas. Since MR guided fusion biopsy technique was not available during 2011–2012, at the time of sampling, we also lack knowledge if the tumor biopsies originate from the dominating intraprostatic lesion. A validated mIHC process in combination with multispectral imaging is a reliable method that demonstrates good overlap with conventional IHC stains and evaluations, reducing the observer variability when quantifying cells.^26,27^ We successfully optimized the mIHC method using PCa biopsies as previously done for colorectal cancer.^26^ Our results demonstrate a correlation between Gleason grade pattern and CD68^+^ cells, as well as a correlation between CD68^+^ macrophages and FOXP3^+^ Tregs in the untreated tissue. Previous studies have demonstrated the same correlations, and this further supports the reliability of the mIHC method used in this study.^18,19^

Another advantage of our study is that investigating five different immune cells simultaneously in the same tissue section saved working hours and valuable tissue material. The digital approach also allowed us to control for unspecific binding, necrotic tissue, and irrelevant areas when calculating cells/mm^2^, as well as making it possible to identify cells stained with multiple markers. We used the digital approach to identify cytokeratin^+^ and FOXP3^+^ tumor cells. The expression of FOXP3 in tumor cells has been linked to poor prognosis in several cancers,^17,22^ and as far as we know, expression of FOXP3 in prostate tumor cells has not yet been investigated. We identified tumor cells with FOXP3 expression, but could not verify any difference in tumor cell FOXP3 expression between treatments. Our mIHC result also demonstrated, among both treated and untreated cases, cells that appeared to express both CD8 and CD20. The software annotated the cell based on the strongest staining, and the weakest stain was ignored. This could be due to overlap between the Opal colors; but, interestingly, a recent study identified T-cells expressing both CD20 and CD8 with high transmigratory and adhesive properties.^37^ To confirm the presence of cells that express both CD8 and CD20 in PCa tissue, further studies need to be performed.

PCa tissue generally has low levels of tumor antigens that trigger the adaptive immune response, and a low number of infiltrating innate immune cells with pro-inflammatory properties. This could explain why immunotherapeutic methods for treatment of PCa have not been successful when used as single treatment.^8,24^ However, one hypothesis is that the tumor tissue damage caused by the conventional PCa treatment can be enhanced if combined with an immunotherapeutic method that amplifies the anti-tumor immunological reactions induced by the damage.^7,23^ In our study, neoadjuvant ADT in combination with RT increased the number of macrophages, indicating a potential benefit from immunotherapeutic methods that reduce the number of M2 macrophages.^7,23^ The increase of Tregs seen after all three treatments indicates that ICI blocking of Tregs has the potential to increase the effectiveness of the treatments.^23^ However, ICI blockers such as anti-PD-1 would also reduce the number of other cells expressing PD-1, such as cytotoxic T-cells, potentially creating an undesirable effect. Moreover, in some areas of the tissue, the therapy response and infiltration of the immune cells may occur quickly and the effect at the end of therapy may be more moderate. The right choice of ICI blocker at the right time point is therefore important.^21,34^

## Conclusion

A validated and standardized mIHC method is a powerful tool for understanding tumor biology and immunology, and hence is valuable in the development of diagnostic, prognostic, and therapeutic methods. Here, we have shown that neoadjuvant ADT in combination with RT results in a higher inflammatory response compared to RT or ADT alone. We conclude that the mIHC method can be a useful tool for investigating infiltrating immune cells in PCa biopsies, and can possibly also act as a guide for understanding which immunotherapeutic approaches may improve the anti-tumor efficacy of current therapeutic approaches in PCa such as neoadjuvant ADT and RT therapy.

## Supplemental Material

Supplemental Material - Infiltrating immune cells in prostate cancer tissue after androgen deprivation and radiotherapy Click here for additional data file. Supplemental Material for Infiltrating immune cells in prostate cancer tissue after androgen deprivation and radiotherapy by Ann Erlandsson, Marie Lundholm, Johan Watz, Anders Bergh, Elitsa Petrova, Farhood Alamdari, Thomas Helleday, Sabina Davidsson, Ove Andren and Firas Tarish in International Journal of Immunopathology and Pharmacology.

## Appendix

### List of abbreviations

ADT: androgen deprivation therapy
ICI: immune-checkpoint inhibitors
mIHC: multiplexed immunohistochemical method
PCa: prostate cancer
RT: radiotherapy
TAM: tumor-associated macrophages
Tregs: regulatory T-cells
