# Supplementary material for: Infiltrating immune cells in prostate cancer tissue after androgen deprivation and radiotherapy
Source: Int J Immunopathol Pharmacol. 2023 Mar 6;37:03946320231158025. doi: 10.1177/03946320231158025 (PMC9996739; doi:10.1177/03946320231158025)

Supplemental Figure 1: Representative serial tissue sections (case B) stained with anti-CD163 or anti-CD68. **a)** CD163$^+$ cells (brown) stained in the standard IHC, **b)** CD68$^+$ cells (brown) stained in the mIHC. Magnification × 20, scale bar 100 μM.

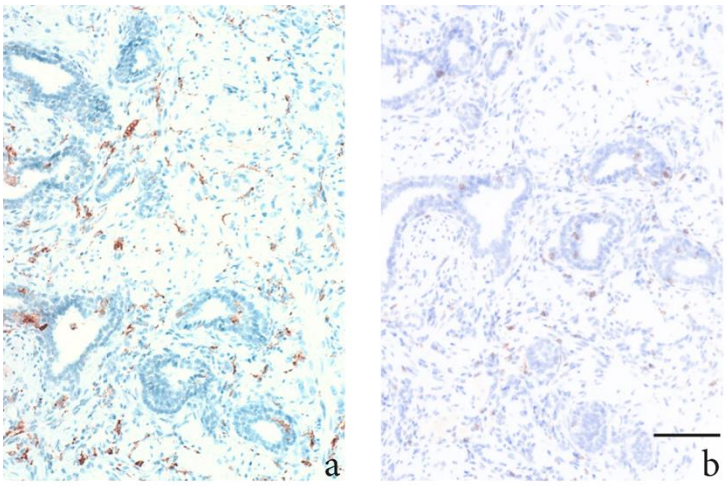

Supplement: Supplemental Material - Infiltrating immune cells in prostate cancer tissue after androgen deprivation and radiotherapy [file sj-pdf-1-iji-10.1177_03946320231158025.pdf]
